# 2017 WSES guidelines on colon and rectal cancer emergencies: obstruction and perforation

**DOI:** 10.1186/s13017-018-0192-3

**Published:** 2018-08-13

**Authors:** Michele Pisano, Luigi Zorcolo, Cecilia Merli, Stefania Cimbanassi, Elia Poiasina, Marco Ceresoli, Ferdinando Agresta, Niccolò Allievi, Giovanni Bellanova, Federico Coccolini, Claudio Coy, Paola Fugazzola, Carlos Augusto Martinez, Giulia Montori, Ciro Paolillo, Thiago Josè Penachim, Bruno Pereira, Tarcisio Reis, Angelo Restivo, Joao Rezende-Neto, Massimo Sartelli, Massimo Valentino, Fikri M. Abu-Zidan, Itamar Ashkenazi, Miklosh Bala, Osvaldo Chiara, Nicola de’ Angelis, Simona Deidda, Belinda De Simone, Salomone Di Saverio, Elena Finotti, Inaba Kenji, Ernest Moore, Steven Wexner, Walter Biffl, Raul Coimbra, Angelo Guttadauro, Ari Leppäniemi, Ron Maier, Stefano Magnone, Alain Chicom Mefire, Andrew Peitzmann, Boris Sakakushev, Michael Sugrue, Pierluigi Viale, Dieter Weber, Jeffry Kashuk, Gustavo P. Fraga, Ioran Kluger, Fausto Catena, Luca Ansaloni

**Affiliations:** 1 General Surgery Papa Giovanni XXII Hospital Bergamo, Bergamo, Italy; 2 Colorectal Unit, Department of Surgery, University of Cagliari, Cagliari, Italy; 3 Unit of Emergency Medicine Ospedale Bufalini Cesena, AUSL Romagna, Romagna, Italy; 4 Trauma TeamGOM Niguarda, Milan, Italy; 5 Department of General Surgery, School of Medicine, University of Milano, Milan, Italy; 6 Department of General Surgery ULSS5 del Veneto, Adria, (RO) Italy; 7 S.S. Annunziata Hospital, Taranto, Italy; 8 Unit of General and Emergency Surgery, Ospedale Bufalini Cesena, AUSL Romagna, Romagna, Italy; 9 Colorectal Unit, Campinas State University, Campinas, SP Brazil; 10 Division of Colorectal Surgery, University of Campinas, Campinas, SP Brazil; 11 General Surgery ASST, Bergamo, Italy; 12 Emergency Department Udine Healthcare and University Integrated Trust, Udine, Italy; 13 Centro Radiológico Campinas, Vera Cruz Hospital, São Paulo, Brazil; 14 Department of Surgery, University of Campinas, Campinas, Brazil; 15 Oncology Surgery and Intensive Care, Oswaldo Cruz Hospital, Recife, Brazil; 16 Department of Surgery Division of General Surgery, University of Toronto, Toronto, Canada; 17 Surgical Department, University of Macerata, Macerata, Italy; 18 Radiology Unit Emergency Department, S. Antonio Abate Hospital, Tolmezzo, UD Italy; 19 Department of Surgery, College of Medicine and Health Sciences, UAE University, Al-Ain, United Arab Emirates; 20 Hillel Yaffe Medical Center Hadera, Hadera, Israel; 21 Trauma and Acute Care Surgery Unit Hadassah, Hebrew University Medical Center, Jerusalem, Israel; 22 Unit of Digestive Surgery, HPB Surgery and Liver Transplant Henri Mondor Hospital, Créteil, France; 23 Department of General and Emergency Surgery Cannes’ Hospital Cannes, Cedex, Cannes, France; 24 Cambridge Colorectal Unit, Cambridge University Hospitals, Cambridge, UK; 25 Division of Trauma & Critical Care University of Southern California, Los Angeles, USA; 26 Department of Surgery, Denver Health Medical Center, University of Colorado, Denver, CO USA; 27 Digestive Disease Center, Department of Colorectal Surgery Cleveland Clinic Florida, Tallahassee, USA; 28 Acute Care Surgery The Queen’s Medical Center, Honolulu, HI USA; 29 Division of Trauma, Surgical Critical Care, Burns, and Acute Care Surgery, University of California San Diego Health Sciences, San Diego, USA; 30 Second Department of Surgery, Meilahti Hospital, Helsinki, Finland; 31 Department of Surgery, Harborview Medical Centre, Seattle, USA; 32 Department of Surgery and Obs/Gyn, Faculty of Health Sciences, University of Buea, Buea, Cameroon; 33 Department of Surgery, Trauma and Surgical Services, University of Pittsburgh School of Medicine, Pittsburgh, USA; 34 General Surgery Department, Medical University, University Hospital St George, Plovdiv, Bulgaria; 35 General Surgery Department, Letterkenny Hospital, Letterkenny, Ireland; 36 Infectious Diseases Unit, Department of Medical and Surgical Sciences, Sant’Orsola Hospital, University of Bologna, Bologna, Italy; 37 Trauma and General Surgeon, Royal Perth Hospital, Perth, Australia; 38 Surgery and Critical Care Assuta Medical Centers, Tel Aviv, Israel; 39 Division of Trauma Surgery, Department of Surgery, School of Medical Sciences, University of Campinas (Unicamp), Campinas, SP Brazil; 40 Department of General Surgery, Division of Surgery, Rambam Health Care Campus, Haifa, Israel; 41 Department of Emergency Surgery, Parma Maggiore Hospital, Parma, Italy

**Keywords:** Colon, Rectum, Cancer, Obstruction, Perforation, Emergency

## Abstract

**ᅟ:**

Obstruction and perforation due to colorectal cancer represent challenging matters in terms of diagnosis, life-saving strategies, obstruction resolution and oncologic challenge. The aims of the current paper are to update the previous WSES guidelines for the management of large bowel perforation and obstructive left colon carcinoma (OLCC) and to develop new guidelines on obstructive right colon carcinoma (ORCC).

**Methods:**

The literature was extensively queried for focused publication until December 2017. Precise analysis and grading of the literature has been performed by a working group formed by a pool of experts: the statements and literature review were presented, discussed and voted at the Consensus Conference of the 4th Congress of the World Society of Emergency Surgery (WSES) held in Campinas in May 2017.

**Results:**

CT scan is the best imaging technique to evaluate large bowel obstruction and perforation. For OLCC, self-expandable metallic stent (SEMS), when available, offers interesting advantages as compared to emergency surgery; however, the positioning of SEMS for surgically treatable causes carries some long-term oncologic disadvantages, which are still under analysis. In the context of emergency surgery, resection and primary anastomosis (RPA) is preferable to Hartmann’s procedure, whenever the characteristics of the patient and the surgeon are permissive. Right-sided loop colostomy is preferable in rectal cancer, when preoperative therapies are predicted.

With regards to the treatment of ORCC, right colectomy represents the procedure of choice; alternatives, such as internal bypass and loop ileostomy, are of limited value.

Clinical scenarios in the case of perforation might be dramatic, especially in case of free faecal peritonitis. The importance of an appropriate balance between life-saving surgical procedures and respect of oncologic caveats must be stressed. In selected cases, a damage control approach may be required.

Medical treatments including appropriate fluid resuscitation, early antibiotic treatment and management of co-existing medical conditions according to international guidelines must be delivered to all patients at presentation.

**Conclusions:**

The current guidelines offer an extensive overview of available evidence and a qualitative consensus regarding management of large bowel obstruction and perforation due to colorectal cancer.

## Background

In 2010, the World Society of Emergency Surgery (WSES) published the guidelines for the management of obstructive left colon cancer [1]. The 2017 guidelines represent both an update and an implementation of the previous edition: the management of perforation and obstruction associated with right-sided colon cancer is also included into the current guidelines.

The relevance of the topic is evident with the help of the following statements:

- Colorectal cancer (CRC) is the third most commonly diagnosed malignancy, accounting for about 1.4 million new cases per year. It represents the third most common cancer in men (746,000 cases, 10.0% of the total) and the second in women (614,000 cases, 9.2% of the total) worldwide; it is the fourth leading cause of cancer death in the world, with almost 700,000 deaths in 2012 [2, 3].
- The incidence of CRC varies by geographic region: in Europe, the incidence is higher than in North America, followed by Oceania, Latin America and Africa. However, the trend of CRC seems to vary according to the Human Development Index (HDI), with a variability parallel to changes in diet, smoke attitude, activity patterns and screening programs. A decreasing rate is reported in North America, Oceania and Europe and in particular in the USA, New Zealand and France; on the other side, an increasing incidence is observed in Latin America, Asia and Eastern Europe [3].

A word of caution must be spent with regards to the increasing incidence of CRC in the population younger than 50 years: this could potentially encourage an update in screening programs [4, 5].

- Complications of large bowel diseases account for 47% of gastrointestinal emergencies [6].
- CRC presents as emergency in a wide range of patients (from 7 to 40% of the total), but the vast majority of reports present a figure of around 30% [6–15].
- Large bowel obstruction (LBO) represents almost 80% (15–30% of CRC) of the emergencies related to CRC, while perforation accounts for the remaining 20% (1–10% of CRC) [7, 12, 16, 17].
- The most common location of CRC obstruction is the sigmoid colon, with 75% of the tumours located distal to the splenic flexure [18].
- Perforation occurs at the tumour site in almost 70% of cases and proximal to the tumour site in around 30% of cases [6, 19, 20].

Management of obstruction and perforation of the colon and rectum secondary to CRC is challenging in terms of clinical severity, diagnostic and therapeutic options and management of septic and oncologic issues.

Focused guidelines lack of evidence and consensus is often limited to short sections within general colon and rectal cancer guidelines edited by Surgical Societies [21–23].

## Materials and methods: consensus conference organisational model

In July 2016, the Scientific Board of the WSES endorsed the President of the Society and the President of the 4th World Congress of the WSES to prepare the Consensus Conference on Colon Rectal Cancer Emergencies (CRCE) focusing on obstruction and perforation.

The Presidents and the six members of the Scientific Secretariat agreed on six key questions to develop the topics for the current guidelines; according to the skills (residency program, work and scientific experience), 12 international experts, affiliates of the WSES, were chosen as Scientific Committee of the Consensus Conference. Each question was developed by members of the Scientific Committee in a variable number from 2 to 4 according to the magnitude of the topic: the Scientific Secretariat members and the Presidents supervised each group.

The documentarist of the Papa Giovanni XXIII Hospital medical library, with the support of the Scientific Secretariat, provided the electronic search in PubMed and EMBASE databases, according to specific key words for each questions, without time or language restrictions (Table 1).

**Table 1:** Questions and MeSH terms

Questions	Key words
Diagnosis	diagnosis, cancer, neoplasm, colon, rectum, bowel, perforation, obstruction, physical examination, radiology, laboratory, abdominal ultrasound, CT scan, colonic enema
Perforation	perforation, cancer, neoplasm, colon, rectum, bowel, tumour perforation, diastatic perforation, faecal peritonitis, treatment, surgery, acute care surgery
Obstruction left	obstruction, left colon, rectum, cancer, neoplasm, surgery, acute care surgery, stent, SEMS, Hartmann’s procedure, colostomy, resection, anastomosis, tube decompression
Obstruction right	obstruction, right colon, rectum, neoplasm, surgery, acute care surgery, stent, SEMS, loop ileostomy, intestinal bypass, resection, anastomosis, tube decompression
Unstable patients	unstable patient, haemodynamic instability, critically ill patient, sepsis, peritonitis, obstruction, cancer, neoplasm, colon, rectum, surgery, acute care surgery, damage control, open abdomen.
Antibiotics	antibiotics, therapy, prophylaxis, colon, rectum, perforation, obstruction, unstable patient, haemodynamic instability, critically ill patient obstruction, bowel, sepsis, peritonitis, surgery, acute care surgery.

The additional bibliography research was developed by each group before starting and updated to May 2017. The research presented at the CC as “in press” has been kept in consideration if published before the final revision of the present guidelines. Each working group, before the CC, developed a focused draft and a variable number of statements along with the level of evidence (LoE) and the grade of recommendation (GoR) for each statement. The 2011 Oxford Classification was used to grade the LoE and GoR (available at https://www.cebm.net/category/ebm-resources/loe/).

The provisional statements and the supporting literature were reviewed by the SS and the Presidents, discussed with the members of each working groups by email and call conferences and modified if necessary.

The Consensus Conference on CRCE has been held in Campinas, Brazil, on May 18, 2017, during the 4th World Congress of the WSES.

The designated member of each working group presented the statements to the audience, along with LoE, GoR and the literature supporting each statement. The audience, represented by 45 experts, voted each statement using a red/green double face card (green, agreement; red, disagreement). The exact agreement/disagreement ratio was not calculated simultaneously to avoid waste of time: for the entire vote, it ranged from 45/0 to 38/7 (18%); despite the small percentage of disagreement, each red card comment was discussed and a final agreement reached among participants.

The agreement required some statement modification, performed by the Presidents and by the Scientific Secretariat; all the statements were eventually reviewed by the WSES board and modified accordingly (Table 6 in Appendix 1).

Further literature published between May and December 2017 was also considered. Clinicians and surgeons must be aware that the present WSES guidelines should be considered as an adjunctive tool for decision and management but they are not substitute of the clinical judgement for the individual patient.

## Results

The results are hereby presented separately as O (obstruction) and P (perforation) when required; otherwise, the statements can be considered valid for both conditions.

### Diagnosis

*Statement 1.1: The clinical presentation is variable, except for lower rectal cancer, in which case digital examination could be diagnostic. Laboratory tests are not specific. Clinical evaluation and laboratory tests have high variability and low specificity; therefore, the escalation to further diagnostic tools, whenever available, is mandatory*. *LoE 3, GoR B.*

#### Obstruction

Large bowel obstruction can present acutely, with colic-like abdominal pain, abdominal bloating and absence of bowel movement and flatus, while vomiting is less frequent than in small bowel obstruction, or subacutely, with gradual development of symptoms, changes in bowel habits and recurrent left lower quadrant abdominal pain. In a series of 150 consecutive patients suffering from acute mechanical bowel obstruction, 24% presented with large bowel obstruction. Absence of passage of flatus (90%) and/or faeces (80.6%) and abdominal distension (65.3%) were the most common symptoms and physical signs [24]**.**

Abdominal examination shows tenderness, abdominal distension and hyperactive or absent bowel sounds.

Previous complaint of bloody stools and passage of blood per rectum, despite the absence of bowel movement, can be associated with colon cancer. A rectal cancer may be palpable as an intrinsic lesion [25, 26]**.**

Laboratory tests are directed at evaluating the electrolyte imbalances, elevated urea nitrogen and metabolic alkalosis that may occur as a consequence of vomiting and dehydration.

#### Perforation

When perforation occurs at the tumour site, peritoneal contamination is usually localised; at the opposite, when perforation is located proximal to the tumour site, the faecal spread results in diffuse peritonitis and septic shock.

In this setting, physical examination reveals an acutely ill patient characterised by fever, tachypnea, tachycardia and confusion.

**Fig. 1:**
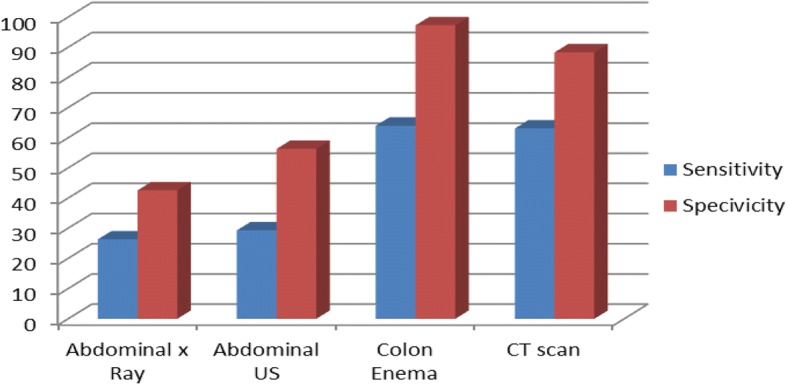
Cumulative diagram for the three items (confirmation, cause, site of LBO) according to imaging study. *US* ultrasound, *CT* computerized tomography

The abdomen may be diffusely tender or may present localised tenderness, guarding, or rebound tenderness. Bowel sounds are usually absent. The toxic symptoms of peritonitis are usually delayed, but are considered an ominous sign [27]**.** Leukocytosis and neutrophilia, elevated amylase levels and lactic acidosis suggest perforation or necrosis [28]. The suspicion of large bowel obstruction or perforation is based on aspecific symptoms, signs and laboratory findings: adjunctive diagnostic tests are mandatory, whenever available (Fig. 1).

*Statement 1.2: (a) In case of clinical suspicion of colon obstruction, computed tomography (CT) scan achieves diagnostic confirmation better than abdominal ultrasound (US), which performs better than abdominal plain X-ray. If CT scan is not available, a water-soluble contrast enema is a valid alternative for identifying the site and the nature of obstruction. (b) In case of clinical suspicion of perforation, abdominal CT scan, which performs better than abdominal US, should achieve diagnostic confirmation. US performs better than abdominal plain X-ray. LoE 3, GoR B.*

Abdominal plain X-ray and abdominal US are screening imaging tests, with the latter representing the more performing alternative. With these results, after adequate training, bedside abdominal ultrasound examination could replace abdominal plain X-ray (Table 2).

**Table 2:** Comparison of imaging studies for confirmation, cause and site of LBO

	Confirmation of LBO obstruction		Cause of LBO		Site of LBO	
	Sensitivity	Specificity	Sensitivity	Specificity	Sensitivity	Specificity
Plain X-ray	74–84% [26]	50–72% [26, 211, 212]	0	7% [212]	0	60% [212]
Abdominal US	88% [211]	76% [211]	0	23% [212]	0	70% [212]
Colonic enema	96% [26]	98% [26]	0	96% [26]	96% [26]	98% [26]
CT scan	93–96% [212, 213]	93–100% [212, 213]	0	66–87% [212, 214]	95% [213]	90–94% [29, 213]

As a consequence, the clinical suspicion of bowel obstruction should be, as a first step, tested by abdominal US or by plain abdominal X-ray when the abdominal US expertise is not available or the US machine is not promptly available.

Despite contrast enema shows acceptable sensitivity and specificity, abdominal CT scan, with high sensitivity and specificity, has the absolute advantage to provide the clinician with an optimal grade of information, in particular regarding the complications of cancer-related LBO. Moreover, it is possible to stage the neoplastic disease and to identify synchronous neoplasms (Table 2). Due to this multifaceted profile, CT scan represents the imaging test of choice in current clinical practice; if CT is available, the water-soluble contrast enema can be considerate obsolete.

When the clinical scenario is suggestive of bowel perforation, abdominal US or abdominal plain X-ray should be used as first screening imaging tests. Bedside abdominal US, performed by a trained physician or surgeon, has higher sensitivity and same specificity of abdominal plain X-ray [29]; moreover, it reduces the mobilisation of a critically-ill patient. One of the limitations of the abdominal US and of the abdominal plain X-ray is the risk of false negatives of pneumoperitoneum, when a small amount of intraperitoneal free air is present, such as in the case of early perforation at the tumour site (Table 3).

**Table 3:** Comparison of imaging studies for confirmation and site of perforation

	Confirmation of perforation		Site of perforation	
	Sensitivity	Specificity	Sensitivity	Specificity
Abdominal plain X-ray	53% [30]	53% [30]	NS	NS
Abdominal US	92% [30]	53% [30]	NS	NS
Colonic enema	NS	NS	NS	NS
CT scan	95% [29]	90% [29]	NS	90% [29]

*NS* not stated

When bowel perforation is suspected, screening imaging tests are represented by abdominal US or abdominal plain X-ray. The literature shows that bedside abdominal US has a higher sensitivity and same specificity with abdominal plain X-ray; moreover, it allows environmental stress reduction to an acutely ill patient [30]**.**

*Statement 1.3: In stable patients, direct visualisation of the site of colonic obstruction should be considered when colonoscopy is available. In this situation, biopsies should be obtained, especially when the deployment of an endoscopic stent is planned. LoE 3, GoR B*

The role of colonoscopy in the setting of the diagnosis of LBO is limited; this is mainly due to its low availability in the emergency setting. The aim of the direct visualisation is to explore the various aetiologies of obstruction. Biopsies and histologic examination of the lesions should be performed when an emergency surgical resection has not been planned or endoscopic stent placement can be expected [18, 21, 31].

*Statement 1.4: In case of diagnosis of perforation at abdominal US or abdominal X-ray in a stable patient, abdominal CT scan should be considered, in order to define the cause and the site of perforation. If there are clear signs of diffuse peritonitis, CT scan should not delay the appropriate treatment. Early involvement of the surgeon is required. LoE 3, GoR B*

Although free air in the peritoneal cavity leads, in the vast majority of cases, to surgical exploration, CT scan examination is suggested if available.

In fact, in few cases of pneumoperitoneum, a conservative management could be attempted, depending on the gastrointestinal perforation site; moreover, there are some cases of pneumoperitoneum which are unrelated to intestinal perforation [32, 33].

Furthermore, CT scan can help the surgeon to foresee the operative scenario, with a better prediction of the resources needed for the intervention [34].

Despite its utility, it must be stressed that CT scan, even when readily available, should never expose the patient to unsafe delays in the appropriate treatment.

*Statement 1.5: There is no specific data regarding staging pathways of CRC presenting as an emergency. CT scan performs better than US in the abdomen and should be suggested for staging in the suspicion of cancer-related colorectal emergencies. CT scan of the thorax is not strictly recommended. LoE 3, GoR B*

The 2017 NCCN guidelines on colon cancer recommend CT scanning for staging of liver metastases from a colorectal primary tumour, given its best accuracy among the available preoperative tests [35].

Evidence to support the indication for routine CT of the thorax is weak: a resolving power of 2–3 mm for lung nodules leads to a sensitivity of 100%, but the specificity is low, with a false positive rate of 34%. On the other side, chest X-ray has a low sensitivity (30–64%), but has a specificity as high as 90% [36].

This data should be kept into consideration also when approaching CRCE: if available, preoperative CT scan of the abdomen should be obtained for staging, while X-ray of the chest may be appropriate for staging of the chest. As previously stated, CT scan for staging should never expose the patient to a safety risk, in terms of time and actions subtracted to the patient's care.

### 2. Management of perforation

*Statement 2.1: When diffuse peritonitis occurs in cancer-related colon perforation, the priority is the control of the source of sepsis. Prompt combined medical treatment is advised. LoE 2, GoR B*

While keeping in mind the caveats of oncologic treatment for patients with perforated CRC, the priority must be directed to immediate patient safety and therefore to treatment of the septic status and to control of the source of sepsis (see Appendix 2).

When free peritonitis, usually related to perforation proximal to tumour site, occurs, patients are at a higher risk of development of septic shock, as compared to patients with a contained collection, which is usually related to perforation at the tumour site for necrosis [6, 19, 20, 37–39].

In cases of contained intra-abdominal collections from perforated CRC, the mortality rate is between 0 and 24%, with an increase to 19–65% in the case of diffuse faecal peritonitis: this difference reaches statistical significance. Despite this finding, the severity of peritonitis, at multivariate analysis, is not an independent factor influencing the in-hospital mortality [6, 20].

Moreover, the in-hospital mortality is related to the site of perforation, varying from 37 to 60% for perforation at the tumour site or proximal to the tumour site, respectively [20].

The treatment of patients with septic shock due to intra-abdominal infection (IAI) is time-dependent; the medical treatment and the source control should be started as soon as possible. The details of the treatment of complicated IAI lie outside the intent of the present study; a number of guidelines are available on this topic [40, 41].

*Statement 2.2: Oncologic resection should be performed in order to obtain better oncologic outcomes.*

- *Perforation at the tumour site: formal resection with or without anastomosis, with or without stoma.*
- *Perforation proximal to tumour site (diastasic): simultaneous tumour resection and management of proximal perforation is indicated. Depending on the colonic wall conditions, a subtotal colectomy may be required. LoE 3, GoR B*

*The surgeon should consider that only a small proportion of patients undergo reversal of a terminal stoma.*

In CRCE, the long-term oncologic outcome can be influenced by an advanced disease and by the higher rate of incomplete preoperative workup. However, in case of perforation, the presence of undiagnosed metastases has a small impact in the treatment strategy.

Keeping in mind that immediate patient safety takes priority, the performance of a standard oncologic resection can lead to similar results, as compared to elective cases.

In the series of Zielinski et al., oncologic features in perforated CRC were obtained: patients were matched comparing free peritonitis versus contained collection; a third group of CRC without perforation was used as a control group (all groups were balanced for demographics and tumour staging). Authors observed a progressive increase in the lymph node harvesting rate across the three groups (free peritonitis, contained collection and no perforation cases); statistical significance was reached only when comparing all perforations versus no perforation (11 versus 16; *p* < 0.001). This significance, however, was not evident when comparing the positive nodes only. For other outcomes, such as completeness of resection, rate of adjuvant therapy and time to adjuvant therapy, the comparison showed no significant difference [6].

Biondo et al., interestingly, observed similar results when comparing patients undergoing emergency surgery for occluded CRC to patients with perforated CRC [19].

Long-term oncologic outcomes were analysed in the same studies: Zielinski and colleagues showed that, when adjusted by excluding the perioperative mortality, overall survival (OS) was similar for free faecal peritonitis, as compared to contained collection and in all perforated compared with non-perforated cases. At the opposite, the inclusion of the perioperative mortality dramatically increases the difference in terms of OS in case of free faecal peritonitis; however, at the multivariate analysis, perforation is not an independent factor for OS [6]. Similar results were obtained by Biondo et al. [19].

As a consequence, in case of perforation at the tumour site:

- For right-sided perforation, a right colectomy should be performed. In case of poor general or adverse local conditions, a resection without anastomosis and terminal ileostomy should be performed.
- For transverse/left-sided perforation: resection with anastomosis, with or without ileostomy, should be attempted. Hartmann’s procedure might be considered, keeping in mind the low rate of stoma reversal. In case of perforation at a distant site from the tumour (generally the neoplasm is in the left colon and the perforation is found in the caecum), a subtotal colectomy should be attempted. The literature reports a better control of postoperative diarrhoea with resection of less than 10 cm of terminal ileum and a distant colon remnant above the peritoneal reflection of at least 10 cm of length [42, 43].

The surgeon managing CRC perforation should decide whether to perform the intestinal anastomosis or to carry out a derivative stoma. There is no evidence of sound quality to guide evidence-based decisions, as specific studies mostly consider heterogeneous groups of perforated and obstructed cancer-related LBO. The rate of anastomotic leak (AL) in right colon resections varies from 0.5 to 4.6% in perforated emergency cases and should be compared with 0.5–1.4% reported for elective surgery; the AL rate after left colonic resection ranges from 3.5 to 30% in emergency versus 5–10% in elective cases [30, 44]**.**

As a general rule, principles of oncologic resection should be followed, always considering the importance of the medical comorbidities and of the septic status on the one side and the aim of a shortened uncomplicated postoperative course in order to allow oncologic staging completion and the start of chemotherapy programs, on the other side.

A word of caution should be spent on the risk of peritoneal carcinomatosis in perforated CRC: a single prospective series is available in the literature. All other series included a small number of patients, with a long data collection time and inclusion criteria, seldom explicated, were heterogeneous (inclusion or exclusion of patients with perforation proximal the tumour site etc.). In 2013, Honoréet al. published a systematic review, concluding that the rate of peritoneal carcinomatosis in perforated CRC ranges between 14 and 54%,with a level of evidence 3b to 4 [45].

### Management of obstruction: left colon (from the distal transverse colon to the anus)

Several options to manage obstructive left colon cancer (OLCC) are available (see Table 4 and Appendix 3).

**Table 4:** Treatment options for OLCC

Main options	Choices among main options	Ancillary manoeuvres among main option and choices
Loop colostomy (C) (bridge to resection or palliation)		
Primary resection with end colostomy: Hartmann’s procedure (HP)		
Resection and primary anastomosis (RPA)	Total/subtotal colectomy (TC)	Intraoperative colonic irrigation (ICI) Manual decompression (MD) Covering stoma
	Segmental colectomy (SC)	
Tube decompression		
Endoscopic colonic stenting by self-expanding metallic stents (SEMS)	Bridge to surgery	
	Palliation	

*Statement 3.1: Loop colostomy (C) versus Hartmann’s procedure (HP)*

*Hartmann’s procedure should be preferred to simple colostomy, since colostomy appears to be associated with longer overall hospital stay and need for multiple operations, without a reduction in perioperative morbidity LoE 2, GoR B.*

*Loop colostomy should be reserved to unresectable tumours (if SEMS is not feasible), for severely ill patients who are too unfit for major surgical procedures or general anaesthesia*.

A stoma provides colonic decompression with minimal surgical trauma, reduces the risk of contamination from an unprepared bowel and allows an intensive resuscitation of the patient and a better staging prior to the definitive treatment.

However, Fielding et al. [46] did not find any differences in the mortality rate between 47 patients treated with loop colostomy and 90 patients who received a primary resection.

A RCT [47] between Hartmann’s procedure (63 patients) and colostomy (58 patients) found no difference in terms of mortality and morbidity rate, recurrence rate and cancer-specific survival between the two surgical approaches. On the other hand, the overall length of hospital stay was shorter in the primary resection (35 days) than in the staged resection group (49 days) (*p* = 0.01).

A Cochrane systematic review [48] considered only other four retrospective cases series and no RCT; therefore, a meta-analysis could not be performed.

Since then, another RCT was published [49]; the authors found a similar impact on mortality and hospitalisation with both surgical techniques.

*Statement 3.2: Hartmann’s procedure (HP) versus resection and primary anastomosis (RPA)*

*RPA should be the preferred option for uncomplicated malignant left-sided large bowel obstruction in absence of other risk factors.*

*Patients with high surgical risk are better managed with HP. LoE 3-GoR B.*

HP remains one of the most common procedures in emergency surgery of the left colon [50–52]. However, the historical concept that a completely clear colon is necessary to avoid AL [53] has been questioned by others [54, 55], and there is now good evidence supporting that the presence of faeces in the large bowel does not influence the rate of anastomotic dehiscence, [56, 57] nor its severity [58].

In recent years, there has been an increasing trend toward a one-stage resection for left-sided obstruction, but unfortunately, no RCTs were conducted comparing HP and RPA; therefore, neither grade A nor B evidence are available, and the choice generally depends on the individual surgeon’s judgement.

The first major report regarding RPA for obstructive cancer came from the Large Bowel Cancer Project (LBCP). The authors reported a mortality of 35% for staged resections and of only 14% for primary resection [46].

Since then, many prospective and retrospective series on RPA in OLCC reported rates of anastomotic dehiscence ranging from 2.2 to 12% [59–65]; these results are almost comparable to the 2–8% rate after elective surgery [56, 57, 66, 67].

Meyer et al. [51] reached different conclusions: they compared HP and RPA performed for OLCC both with curative and palliative intent. Despite the significantly higher preoperative risk within the HP group, postoperative mortality rate was lower as compared to the RPA group, both for curative (7.5 versus 9.2%; *p* value reported as not significant) and palliative procedures (33 versus 39%; *p* value reported as not significant). The limit of this study was the high number of participating institutions (309), which were also very heterogeneous in terms of intensity of care, spanning from regional to university hospitals.

The main advantage of RPA is to avoid a second major operation, which is associated to a morbidity rate of 20–50% and a dehiscence rate of 2–7% [68–72].

Furthermore, it should be considered that the majority of stomas (up to 90%) created during HP for CRC do not get reversed, due to necessity of adjuvant treatment and/or disease progression [62, 73].

In favour of RPA, it has also been postulated that this choice may result in long-term survival benefits, although evidence on this aspect is weak [65].

These unquestionable advantages of RPA must be counterbalanced by the potentially catastrophic situation resulting from AL in a fragile patient. For this reason, many parameters, related to both the surgeon and the patient, should be taken into account before deciding to perform a colo-colonic or colo-rectal anastomosis [63, 64, 74]. Historically, two main elements prevent anastomotic dehiscence: a tension-free anastomosis and good blood supply to the anastomotic rim; despite the single surgeon’s experience may play a pivotal role in the evaluation of these parameters, evidence exists regarding the validity of the assessment of the anastomotic blood supply using intraoperative near-infrared indocyanine green [75, 76]. Risk stratification is the cornerstone of patient’s selection. The Association of Coloproctology of Great Britain and Ireland (ACPGBI) identified four important predictors of outcome—age, ASA grade, operative urgency, and Dukes’ stage [64]; others showed similar results [63, 74].

The experience and subspecialty of the surgeon also seem to be important factors in surgical decision. It has been demonstrated that primary anastomosis is more likely to be performed by colorectal rather than general surgeons, and by consultants rather than unsupervised trainees, with lower rate of anastomotic dehiscence and mortality [46, 74, 77–80].

Keeping in mind these considerations, HP could be more appropriate for patients deemed to be at high risk and when they are managed in an emergency setting by unspecialised surgeons.

*Statement 3.3: RPA: the role of diverting stoma*

*There is no evidence supporting that a covering stoma can reduce the risk of anastomotic leak and its severity. LoE 4-GoR C*

Unfortunately, there are very few data and no RCT comparing the use of diverting stoma versus no use of diverting stoma after surgery for OLCC; therefore, very weak recommendations can be drawn.

Kube et al. [81] analysed the results of 743 patients who underwent emergency radical surgery for OLCC. Of these, 30% had HP, 58% RPA and 12% RPA and covering stoma.

The morbidity and hospital mortality did not differ significantly between the groups, and the addition of a protective stoma did not affect the rate of anastomotic dehiscence (7 and 8% respectively), or the rate of re-operation (5.6 versus 5.7%).

We may postulate that a protective stoma does not reduce the rate of AL, but the rate of AL requiring re-operation [82]. A leak originating from an intraperitoneal anastomosis is likely to cause diffuse peritonitis and therefore mandates a reoperation. For this reason, the role of diverting stoma after resection and primary anastomosis for OLCC seems limited.

*Statement 3.4: Total colectomy versus segmental colectomy*

*In absence of caecal tears/perforation, evidence of bowel ischemia or synchronous right colonic cancers, total colectomy should not be preferred to segmental colectomy, since it does not reduce morbidity and mortality and is associated with higher rates of impaired bowel function. LoE 2, GoR B.*

Total colectomy (TC) with ileo-rectal anastomosis was proposed as an alternative procedure to avoid a stoma and at the same time to overcome the problems related to a distended unprepared colon [83–85]. This operation has an absolute indication when obstruction has determined a right colonic ischemia, caecal tears or perforation, or when synchronous proximal malignant tumours are present [21].

Major disadvantages of TC are represented by a technically challenging procedure, prolonged operative time and poor functional results, with many patients complaining of diarrhoea and possibly developing electrolyte disturbances [84, 86].

A single RCT, the SCOTIA (Subtotal Colectomy versus On-Table Irrigation and Anastomosis) trial was published [86]; 91 patients from 12 different centres were randomised to total/subtotal colectomy (47 patients) versus segmental colectomy with on-table lavage (44 patients). The authors found no differences in terms of morbidity and mortality, but significantly worse functional results after TC.

*Statement 3.5: Intraoperative colonic irrigation (ICI)* versus *manual decompression (MD)*

*ICI and MD are associated with similar mortality/morbidity rate. The only significant difference is that MD is a shorter and simpler procedure. Either procedure could be performed, depending on the experience/preference of the surgeon. LoE 2-GoR B*

There was only a RCT that compared ICI (24 patients) with MD (25 patients) in OLCC [87]. They concluded that MD is shorter and simpler than ICI and offers similar results in terms of mortality, morbidity and AL rates. However, the power of this study was low.

A systematic review published in 2009, which included the above-mentioned RCT, one prospective comparative trial and 5 prospective descriptive case series, concluded that, although the power of the studies was poor and a large-scale prospective randomised trial is desirable, no statistical significance could be shown between the two procedures [88].

*Statement 3.6: RPA: the role of laparoscopy*

*The use of laparoscopy in the emergency treatment of OLCC cannot be recommended and should be reserved to selected favourable cases and in specialised centers.*

*LoE 4-GoR C*

Traditionally, CO has been considered an absolute contraindication to laparoscopy, because of the high-risk patient profile and the level of operative technical difficulties due to dilated and vulnerable bowel [89].

However, with the diffusion of colo-rectal laparoscopy and increasing experience, some limited series became available with favourable results [90, 91], but no randomised trials have been produced.

Ballian et al. [92] evaluated the role of laparoscopy for emergency restorative colectomy using the American College of Surgeons National Surgical Quality Improvement Program (ACS NSQIP) database. They found that less than 10% of patients with OLCC were managed laparoscopically with colon resection and primary anastomosis, with comparable rates of morbidity and mortality, but faster recovery.

A systematic review published in 2014 analysed the results of 47 studies on laparoscopy in emergency colorectal surgery, but most of them regarded acute presentation of IBD or diverticular disease, while only a small number presented data on OLCC [93].

*Statement 3.7: Tube decompression (TD)*

*TD can be a valid alternative option as BTS for high-risk OLCC. LoE 4-GoR C*

Transanal TD is a minimally invasive endoscopic procedure that may allow the decompression of an obstructed colon in order to safely delay elective surgery with RPA. Despite the appeal for this bridge to surgery technique, unfortunately only few data is available.

Efficacy and safety of TD have been reported [94–102], with 80 to 100% rate of technical success and 72.5 to 100% rate of clinical success. Complications, such as perforation, are infrequent (incidence ranging from 0 to 10%) and may be caused by the pressure of the tip of the tube against the colonic wall.

However, there is lack of trial-based evidence to confirm the usefulness of TD and its efficacy in terms of short- and long-term outcomes.

Theoretically, TD has some advantages over self-expandable metallic stent (SEMS): the colon can be cleaned by lavage through the tube; tumour manipulation is minor and costs are contained. However, there are no randomised trials but only one retrospective study that compared these two techniques and did not show significant differences [103].

Despite these results appear promising, the available level of evidence is suboptimal, and therefore, no conclusions can be drawn.

*Statement 3.8: Palliation: SEMS* versus *colostomy*

*In facilities with capability for stent placement, SEMS should be preferred to colostomy for palliation of OLCC since it is associated with similar mortality/morbidity rates and shorter hospital stay. LoE 1-GoR A*

*Alternative treatments to SEMS should be considered in patients eligible to a bevacizumab-based therapy. Involvement of the oncologist in the decision is strongly recommended. LoE 3-GoR B*

Endoscopic stent placement was initially introduced in the palliative treatment of obstructive rectal [104] or recto-sigmoid cancer [105].

The development of SEMS, which can be introduced through a colonoscope, allowed to extend their use to a range of scenarios of CO [106, 107], not only with palliative intent to avoid a stoma, but also with the aim of transforming an emergency surgical operation into an elective procedure, and od reducing morbidity, mortality and stoma rate [108].

Several RCTs, case-matched studies and retrospective series have been published, but results are controversial.

We found five RCT comparing colostomy versus SEMS for palliation of malignant CO [109–112]; one of them was an update of a previous RCT [113].

Xinopoulos et al. [109] randomised 30 patients. A stent was successfully placed in 14/15 (93.3%) randomised to stenting, and CO was permanently resolved in eight of them (57%). There was no mortality related to the procedure in both groups. Mean survival was 21.4 months in SEMS group and 20.9 months in C group. Mean hospital stay was significantly higher in C group, and costs were comparable. The authors concluded that SEMS placement represents a good alternative to colostomy, providing a better quality of life for the patient, without the psychological repercussions of a colostomy, and it appears to be cost-effective.

Fiori et al. [110] randomised 22 patients: in both groups, the mortality was 0% and the morbidity was similar. SEMS group had shorter time to oral intake, restoration of bowel function, and hospital stay.

Some years later, the same group published the long-term results [113]: mean survival was 297 days (125–612) with SEMS and 280 days (135–591) in patients with stomas (*p* = n.s.). There was no mortality related to the procedures. Patients with stomas found them unacceptable, and the same feelings were present in their family members. On the contrary, none of the patients with stents or their family members reported any inconveniences related to the procedure.

The Dutch Stent-in I multicenter RCT [111] was terminated prematurely after enrolling 21 patients; the decision was taken after the incidence of four stent-related perforations among 10 patients enrolled for SEMS (in particular occurring12, 12, 44 and 106 days after stent placement), resulting in three fatal events.

No clear explanation for such a high perforation rate was retrieved; the authors suggested that changes made in the design of the stents (WallFlex, Boston Scientific Natick, MA), which have a larger diameter of the proximal end (30 mm) and are made of braided nitinol instead of stainless steel, might have had a role in the aetiology of the perforation. However, other subsequent series in which the Wallflex stent was used reported a perforation rate of around 5% [114–116], which is in line with commonly observed figures with other SEMS [116].

A more recent RCT [112] enrolled 26 patients in the SEMS group and 26 in the surgery group, with the primary aim to assess the quality of life through a validated questionnaire. Stent insertion was successful in 19 cases (73%), while the remaining patients required a stoma. There were no stent-related perforations. The SEMS group had significantly reduced procedure time (*p* = 0.014) and post-procedure stay (*p* = 0.027). Thirty-day mortality was 8% in the SEMS group and 15% in the surgery group (*p* = 0.668). There was no difference in median survival (5.2 versus 5.5 months), but the surgery group had significantly reduced quality of life.

Several meta-analyses [117–120], pooling data from RCT and from prospective non-randomised or retrospective studies, showed results in favour of stent placement.

According to the available RCTs [109, 112, 113], palliation with the use of SEMS could affect the OS indirectly, by increasing the risk of local complications, such as tumour site perforation, and therefore requiring the interruption of chemotherapy [118, 119].

A correlation between chemotherapy with bevacizumab and stent-related perforation has been noticed [116, 121].

A recent meta-analysis, including 4086 patients from 86 studies, confirmed an increased risk of perforation in patients with bevacizumab treatment, as compared to absence of concomitant chemotherapy (12.5 versus 9.0%) [122].

For this specific reason, the recently published European Society of Gastrointestinal Endoscopy (ESGE) Clinical Guidelines do not recommend the use of SEMS in patients who are being treated with or are expected to be commenced on antiangiogenic drugs [123].

*Statement 3.9: Bridge to surgery*: *SEMS and planned surgery* versus *emergency surgery.*

*SEMS as bridge to elective surgery offers a better short-term outcome than direct emergency surgery. The complications are comparable, but the stoma rate is significantly smaller. LoE 1-GoR A*

*Long-term outcomes appear comparable, but evidence remains suboptimal; further studies are necessary.*

*For these reasons, SEMS as BTS cannot be considered the treatment of choice in the management of OLCC, whilst it may represent a valid option in selected cases and in tertiary referral hospitals. LoE 1-GoR B*

SEMS as BTS allows timely resolution of the obstruction before definitive surgical treatment, giving the possibility of an elective surgical procedure.

For this reason, soon after the introduction of the new devices [105, 124], BTS with SEMS has been considered a pivotal change in the management of colonic obstruction [106] and has been rapidly implemented in clinical practice, although solid scientific evidences were still missing.

In 2012, Zhang et al. [125] performed a meta-analysis of eight studies, including six retrospective studies. Pooled data showed impressive results in favour of stent placement.

These extremely favourable results, however, were not confirmed by other studies, which reported a worrisome trend towards a stent-driven enhanced risk of oncologic recurrence [126–128].

When adjunctive results from randomised controlled trials became available, the overall efficacy of BTS with SEMS appeared to be less definite than previously reported.

Considering a total of seven trials [111, 129–134], three were prematurely terminated for the following reasons: very high morbidity rate in the SEMS BTS group [111], very high morbidity rate in the ES group[130] and high technical failure rate with SEMS [131], respectively.

Summarising the results of these trials, the following main findings arise.

Firstly, the rate of clinical success, which was originally reported to be over 90%, dropped to a mean of around 70%. Secondly, short-term results (in particular postoperative morbidity and mortality, length of hospital stay) appeared comparable between ES and BTS with SEMS. This was also confirmed by the most recently published RCT [134]. The trial was designed to recognise a 20% decrease in morbidity in the stent group as compared to the ES group, but in fact, complications occurred in 51.8% of SEMS group patients and 57.6% of direct surgery group (*p* = 0.5).

On the other hand, all the RCTs have shown that the use of SEMS is related to a reduction in the rate of stomas.

Moreover, the use of SEMS increments the odds of laparoscopic resection. The so-called endo-laparoscopic approach consists in endoscopic stent followed by laparoscopic elective surgery [129, 135, 136].

In the RCT by Cheung et al. [129], all patients undergoing direct surgery had an open approach, while 60% of patients in the SEMS group were managed laparoscopically.

All these considerations have been confirmed by comprehensive data from different meta-analyses [137–143] it can therefore be affirmed that SEMS as BTS provides better short-term outcomes than direct ES.

The oncologic issues related to this approach remain uncertain, and this represents a relevant field of future research.

Analysis of available data from RCT considering long-term outcomes [130, 133, 134, 144, 145] does not show significant harmful effects in OS with SEMS use; however, three of them [130, 133, 145] have reported a tendency towards a diminished disease-free survival (DFS). In particular, Alcantara et al. [130]reported a rate of recurrence as high as 53.3% (8/15) after SEMS versus 15.4% (2/13) after ES.

Moreover, a recent case-control study suggested that SEMS placement might have a critical negative impact on the tumour anatomical site; the authors noticed a significantly higher percentage of tumour ulceration, perineural invasion and lymph node invasion in the SEMS group as compared to the surgery-only group [126].

The main problem related to a potential augmented risk of recurrence after SEMS is the risk of perforation, which is reported in up to 13% of cases. In addition, Pirlet et al. described a peculiar analysis on postoperative pathology, showing that an undetected perforation was present in almost 27% of SEMS [131]. Risk of perforation constitutes a major concern, as underlined by a post hoc analysis of one RCT, in which the 4-year DFS rate was 0% in patients with a stent-related perforation, versus 45% in patients without perforation [145].

Although worrisome to a certain extent, these results come from studies with small number of patients and with an overall short follow-up time to guide definitive conclusions.

Matsuda et al. performed a meta-analysis to specifically investigate the long-term outcomes of SEMS [142]: 11 studies were included, with a total of 1136 patients, but only two of them were RCT, while two were prospective series and seven retrospective.

OS was reported in all studies (3-year OS in 3 of them), while DFS and recurrence in six and eight studies, respectively. Pooled data showed no significant difference between SEMS as a BTS and ES groups neither in OS (RR = 0.95; 95% CI 0.75–1.21; *p* = 0.66), nor in DFS (RR = 1.06; 95% CI = 0.91–1.24; *p* = 0.43) and recurrence rate (RR = 1.13; 95% CI 0.82–1.54; *p* = 0.46).

Similar results were presented in the meta-analysis from Ceresoli et al. [146]. Seventeen studies (5 RCTs, 3 prospective and 9 retrospective comparative cohort studies), for a total of 1333 patients, were included in the analysis. No significant differences were noticed in recurrence rate (RR = 1.11 95% CI 0.84–1.47, *p* = 0.47), 3-year mortality (RR = 0.90 95% CI 0.73–1.12, *p* = 0.34) and 5-year mortality (RR = 1.00 95% CI 0.82–1.22, *p* = 0.99). No differences were found among randomised and observational studies.

As stated by the authors, both these meta-analyses have a great limitation related to the quality of the considered studies: none of the included studies was designed for long-term follow-up, median follow-up times were generally short and heterogeneous and survival rates were estimated with the Kaplan–Meier method rather than with observed events.

For these reasons, although encouraging, these results must be considered with extreme caution. A “non-inferiority” RCT with survival as primary end point would be the appropriate method to correctly investigate long-term outcomes after SEMS as BTS versus ES.

*Statement 3.10: Extraperitoneal rectal cancer.*

*Locally advanced rectal cancers are better treated with a multimodal approach including neoadjuvant chemoradiotherapy. LoE 1-GoR A*

*In case of acute obstruction, resection of the primary tumour should be avoided and a stoma should be fashioned, in order to permit a correct staging and a more appropriate oncologic treatment.*

*Transverse colostomy seems to be the best option, but other modalities can be considered. SEMS is not indicated.*

Extraperitoneal rectal cancers have particular features, which deeply influence the management of obstructive disease.

It has to be considered that a rectal cancer producing an obstruction invariably represents a locally advanced disease. For this reason, if curative resection is judged to be possible, elective surgery should be preceded by neoadjuvant chemotherapeutic treatment [147–150]. The direct consequence of this consideration is that, in case of obstructive emergency, the surgical procedure of choice has to be restricted to techniques aiming to solve the obstruction and to permit a timely initiation of multimodal therapies. Furthermore, the surgical procedure should provide a long-term solution, allowing to conduct the patient through the entire duration of neoadjuvant treatment, until the execution of definitive surgery, and avoiding interferences with the therapeutic schedules and final oncologic result.

- Decompressive stoma versus SEMS

No comparative studies between endoscopic stenting and faecal diversion are available.

However, use of SEMS in low rectal cancer has been linked to chronic pain and tenesmus [102] and a consequential worsening of quality of life. Radiation and chemotherapy, determining tumour necrosis and shrinkage, may favour the development of complications such as migration and perforation that might compromise the final oncologic results.

Moreover, it should be considered that a stoma will be fashioned in any case at the time of surgical resection, either in the case of abdominal-perineal resection or in the case of low anterior resection, where a diverting temporary stoma is highly recommended [151–153].

All these being considered, it is always preferable to manage rectal obstruction with a stoma; the surgeon should plan the future surgical resection and choose the stoma type and location accordingly.

- Loop ileostomy versus loop colostomy versus end colostomy

In essence, and in an ideal situation, the type and location of the emergency stomas should correspond to the type and location of future diverting or definitive stoma.

Previous studies [151, 154, 155] and a recent meta-analysis [156] of trials comparing loop ileostomy versus loop colostomy after elective anterior resection showed better results after loop ileostomy.

Despite this, in case of an emergency rectal obstruction and a planned future anastomosis, a loop ileostomy is a viable option only if the obstruction is incomplete or the ileocaecal valve is patent; otherwise, colonic distension would not be solved. In presence of a complete obstruction and a competent ileocaecal valve, a colostomy is mandatory. Scientific evidence to guide the choice of type a location of the emergency colostomy is limited.

As stated above, the choice of type (end or loop) and site (transverse versus sigmoid colon) of colostomy should be tailored on the individual patient considering the planned definitive treatment.

Limited to patients at high risk for general anaesthesia, a loop left side colostomy could be fashioned under local anaesthesia and intravenous sedation via left side skin incision (the so-called trephine stoma) [157].

A widely used practical approach consists in a right-sided loop transverse colostomy. This is preferred over a sigmoid colostomy because it can be left in place to protect the anastomosis after the planned surgical resection, it is easier to be fashioned due to the mobility of the transverse colon, it avoids the risk of damage to the marginal arcade and it does not alter the left abdominal region in case a permanent end colostomy becomes necessary at the time of definitive surgical resection. When an abdominal-perineal resection is predictable, an end sigmoid colostomy could be a valid alternative [158].

## Management of obstruction: right colon

Different surgical and non-surgical procedures could be offered in the case of obstructive right colon cancer (ORCC) (Table 5); however, right colectomy with anastomosis has been considered safe, and the literature is poor or absent in comparing theoretical options.

**Table 5:** Treatment option for ORCC

Main options	Choices among main options
Resection and anastomosis	
Resection and anastomosis with proximal stoma creation	
Resection and stoma creation	
Stoma creation	
Intestinal internal bypass	
Endoscopic stent placement	Palliative/definitive
	Bridge to surgery

*Statement 4.1.*

*In case of right-sided colon cancer causing acute obstruction, right colectomy with primary anastomosis is the preferred option. A terminal ileostomy associated with colonic fistula represents a valid alternative if a primary anastomosis is considered unsafe. LoE 2-GOR B*

The literature regarding ORCC is definitely less extensive than for OLCC, and this may be related to favourable anatomical reasons and limitation of alternatives to surgery, which lead to the predominance of RC with primary anastomosis as the treatment of choice. Several anatomical reasons can explain this phenomenon: firstly, the hepatic flexure is easier to mobilise as compared to the splenic flexure; secondly, the mobility of the small bowel allows the surgeon to perform the ileo-colic anastomosis without adjunctive surgical manoeuvres; thirdly, the ileo-colic anastomosis benefits from an optimal blood supply, as compared to some critical zones of the left colon and rectum, whose blood supply is dependent on the patency of the marginal arcade and the hypogastric vessels. Segmental right colon resection is extremely rare, due to the reduced length of the right colon and to the common compromise of the caecum [102].

Ileostomy creation should be balanced with the risk of electrolyte imbalance; moreover, alternatives to surgery are scarce and this is related to the considerable technical difficulty of performing operative manoeuvres, stenting or tube decompression, once reached the right colon.

For these reasons, RC with primary ileo-colic anastomosis reprsents the option of choice in ORCC, despite the fact that patients are usually older and with a more advanced locoregional disease than patients with OLCC [159].

As previously mentioned, results from the literature are often mixed inside the broader class of colon emergencies. The rate of AL in the emergency RC is considered to be acceptable, especially when compared to left colon resection with primary anastomosis and to elective cases. However, retrospective studies reached heterogeneous results: Lee et al. reported no differences in the mortality or leak rate between patients with right-sided and left-sided lesions (mortality: 7.3 versus 8.9%; leakage: 5.2 versus 6.9%) [160, 161]; furthermore, in other reports, the AL rate in RC ranges from 0.5 to 4.6% in perforated emergency cases and it should be compared to 0.5–1.4% reported for elective surgery. The interpretation of the comparison of AL risk between ORCC and OLCC may be cryptic: the high heterogeneity of results in several studies, in which the AL rates range for OLCC from 3.5 to 30% for emergency cases and from 5 to 10% for elective cases, underlines this issue [27, 30, 44, 162]**.**

No relevant reports exist comparing the AL rate, the overall complications and the surgery-related mortality between RC and ileo-colic anastomosis with or without proximal loop ileostomy.

*Statement 4.2: For unresectable right-sided colon cancer, a side-to-side anastomosis between the terminal ileum and the transverse colon (the internal bypass) can be performed; alternatively, a loop ileostomy can be fashioned. Decompressive caecostomy should be abandoned. LoE 2-GOR B*

No relevant studies compare the possible options to manage ORCC with unresectable right colon cancer: internal bypass should be preferred to loop ileostomy. Surgical caecostomy should be abandoned for the high rate of malfunction and complications: the role of caecostomy could be reserved, via percutaneous technique, to an extremely small group of fragile patients [23, 163–167]**;** the use of covered expandable stent over previous malfunctioning percutaneous catheter has been reported [168].

*Statement 4.3:*

*SEMS as bridge to elective surgery for ORCC is not recommended. It may represent an option in high-risk patients. LoE 2-GOR B*

We already fully analysed SEMS as BTS in the OLCC section.

The experience for ORCC especially looks into feasibility and safety. In a recent multicenter retrospective study [169], the most appropriate treatment approach for patients with ORCC was evaluated, by comparing short-term postoperative outcomes and long-term oncologic outcomes after ES (emergency surgery), and BTS short-term and long-term outcomes in the BTS group were not inferior to those in the ES group. Right colon stenting is considered technically challenging and future comparative studies are needed for the development of an evidence-based recommendation for clinical decision-making [170].

*Statement 4.4:*

*In a palliative setting, SEMS can be an alternative to emergency surgery (ES) for obstruction due to right colon cancer. LoE 3, GOR B*

The use of stents in patients with incurable large-bowel obstruction presents a number of advantages and some benefits in terms of quality of life, such as faster return to oral diet, decreased stoma rates and reduced post-procedure stay [112].

Shim et al. evaluated the use of a new self-expanding through-the-scope (TTS) double colonic stent in the palliative management of patients with inoperable proximal malignant CO. He concluded that placement of these new self-expanding through-the-scope double colonic stents for the management of inoperable proximal malignant CO is a feasible, effective, and safe form of palliative treatment for the prevention of stent migration and tumour ingrowth [171].

## Unstable patients

*Statement 5.1: A patient with perforation/obstruction due to colorectal cancer should be considered unstable and therefore amenable for damage control treatment, if at least one of the following items is present:*

- *pH < 7.2*
- *Core temperature < 35 °C*
- *BE < − 8*
- *Laboratory/clinical evidence of coagulopathy*
- *Any signs of sepsis/septic shock, including the necessity of inotropic support*

### LoE 2-GoR C

Obstruction or perforation for CRC could lead to instability of the clinical conditions: some reasons can be recognised in fluid and electrolyte imbalance, bacterial overgrowth with translocation across the intestinal wall, peritonitis and pre-existing comorbidities.

When facing this scenario, the emergency physician, the surgeon, and the anesthesiologist should keep in mind the appropriateness of the damage control philosophy. Correct patient selection is crucial to maximise the benefit of damage control surgery (DCS), avoiding at the same time its overuse. Suboptimal evidence is available for non-trauma patients; however, as in trauma setting, the clinical picture may be represented by a mix of patient's factors (comorbidities, medical therapies), physiologic parameters (hypothermia, acidosis, coagulopathy, early organ dysfunction) and treatment/iatrogenic factors (magnitude and quality of resuscitation, time spent in surgery); when these items are present simultaneously, they could depict a patient with a severe physiological derangement and thus an “unstable patient” in the setting of non-traumatic acute care surgery [172–174].

A new definition of septic shock has been recently proposed [175], as a persisting hypotension requiring vasopressors to maintain MAP over 65 and a persistent lactate level > 2 despite adequate volume resuscitation. Several scores have been developed: the APACHE score is validated for use within an ICU setting [176], the MEDS allows to stratify septic patients presenting to the ED [177], the SOFA score allows a calculation of both the number and the severity of organ dysfunctions [178], PIRO is a staging system [179] and the quick SOFA (qSOFA) is validated to identify adult patient with suspected infection who are likely to have poor outcomes [180]. WSES Sepsis Severity Score [181] has been recently validated as a practical clinical score for patients having complicated intra-abdominal infections. A score of 5.5 is predictive of mortality with a sensitivity of 89.2% and a specificity of 83.5% [181]. Each of these scores can be successfully applied, depending on the experience and preference of the clinician involved: the adoption of an institutional score, among the validated ones, should be encouraged in order to allow early recognition of unstable patients.

*Statement 5.2: Damage control should be started as soon as possible, in rapid sequence after resuscitation. LoE 2-GoR C*

The damage control (DC) concept has been extended from trauma surgery to non-trauma surgical emergencies taking into account that, despite different aetiologies, the physiological derangements experienced by the patient are comparable. Often, in emergency general surgery, the physiologic exhaustion is driven by sepsis or septic shock, as in perforated patients or in patients with a closed loop colonic obstruction induced by cancer determining a bacterial overgrowth in the obstructed segment, with mucosal barrier breakdown and subsequent bacterial translocation. In a retrospective analysis on 291 non-trauma patients, Person et al. [172] demonstrated that peritonitis was the most common indication for abbreviated laparotomy in accordance to DC philosophy and that 29% of subjects who underwent DC was unstable on admission to the emergency department.

The key in the preoperative phase is to correctly identify patients who can benefit from DC and thus to consider early and expedite surgery.

Some trigger points to dictate DC in emergency general surgery may be borrowed from the trauma setting, such as hypothermia (core temperature below 35 °C), metabolic acidosis (pH < 7.2; BD > 8) and clinical or laboratory evidence of coagulopathy [173]. In a recent retrospective review of non-trauma emergencies, Becher et al. [174] confirmed that the aforementioned conditions, in association with signs of sepsis or septic shock, age ≥ 70 years and multiple comorbidities identify a profile of decompensated patient who benefits from the DC approach. This evidence supports previous results, published by Subramanian et al. [182], which demonstrated that DC is safe and effective if applied in elderly non-trauma patients with diminished physiological reserve due to intra-abdominal catastrophes.

Differently from a trauma setting, the application of DC in non-trauma surgical emergency requires an initial period of resuscitation, before surgical intervention, in order to prevent haemodynamic instability on induction of anaesthesia. Few hours are necessary to re-establish adequate—and not necessarily optimal—organ perfusion and to start broad-spectrum antibiotic therapy [183].

Azuhata and coworkers [184], in a prospective observational study, demonstrated that the time from admission to initiation of surgery for source control is a critical determinant of 60-day survival in patient with GI perforation with associated septic shock, showing a survival rate of 0% when time to initiation of surgery was greater than 6 h.

Trying to achieve the aims of central venous pressure (CVP) of 8–12 mmHg, mean arterial pressure (MAP) ≥ 65 mmHg and central venous oxygen saturation (ScvO_2_) ≥ 70% within 6 h from the admission, the resuscitation phase should utilise goal-directed methods to guide treatments [185–187].

In addition to volume resuscitation, vasoactive medications may be required, being noradrenaline the first-line agents and adrenaline the second-line agent, while the use of dopamine should be restricted. The administration of solutions containing bicarbonate is not recommended to correct hypoperfusion-induced lactic acidosis, unless pH < 7.15. The alkalinizing agents may be needed in patients with severe acidosis (pH < 7.15) secondary to catecholamine receptor resistance-induced hypotension [183].

Once in the operating theatre, the aim of DC is to obtain source control, while the anatomical reconstruction and abdominal closure must be considered as secondary goals, to be deferred to a secondary procedure after physiological normalization. The precise technical procedure used to achieve source control of sepsis will vary depending on the local situation, the pathology encountered and the degree of physiological derangement.

*Statement 5.3: If the patient is unstable, definitive treatment can be delayed. LoE 2-GoR C*

#### Right-sided obstruction

*Right colectomy with terminal ileostomy should be considered the procedure of choice.*

*Severely unstable patients should be treated with a loop ileostomy.*

#### Right-sided perforation

*Right colectomy with terminal ileostomy should be considered the procedure of choice.*

*If an open abdomen has to be considered, the stoma creation should be delayed.*

*Right colectomy with ileo-colic anastomosis could be performed if no significant increase in operative time is required and good bowel vascularisation is present and expected in the perioperative time.*

#### Left-sided obstruction

*Hartmann’s procedure should be considered the procedure of choice. Severe unstable patients should be treated with a loop transverse colostomy.*

#### Left-sided perforation

*Hartmann’s procedure should be considered the procedure of choice. If an open abdomen has to be considered, the stoma creation should be delayed.*

For patient presenting acutely, the prognosis is poorer as compared to patients presenting under elective admission. Emergency patients are older and have more advanced tumours. Colon obstruction causes volume depletion and electrolyte disorders, while perforation may induce generalized peritonitis. These coexisting factors may lead to patient instability, represented by metabolic impairment, coagulopathy and signs of sepsis/septic shock. All these factors contribute to the alteration of the upstream of the intestinal wall that explain the high risk of AL in the emergency setting, ranging from 4 to 13% [30, 161].

Literature data regarding outcomes after emergency abdominal surgery in patients with advanced cancer suggests that patients experience a high burden of complications and high postoperative mortality after these interventions [188, 189].

As a general principle, all efforts should be made to resect the tumour at the index operation, but this concept has to be balanced with caveats of Damage Control Surgey: exhausted patients should undergo only the procedures they can tolerate, and usually this corresponds to technically easy and rapidly performed interventions, representing life-saving procedures.

Therefore, surgical options for complicated colorectal cancer depend primarily on the location of the tumour, comorbidities of the patient and degree of their clinical status derangement at presentation.

For right-sided lesions, a definitive treatment RC and ileocolic anastomosis can be considered, on the basis of a non-significant increase in operative time as compared to staged procedures; however, surgeon should remember that the AL rate and the mortality for resection in emergency is higher than in elective cases (0.5–4.6 versus 0.5–1.4%; 7 versus 5.3%): although no specific data is available, a higher rate of AL is reasonably expected in the critical scenarios.

If the clinical condition suggests to avoid the creation of an anastomosis, a terminal ileostomy is recommended. The transverse colon can be stapled or a mucous fistula can be occasionally created.

If an open abdomen (OA) has to be considered, stoma creation should be avoided and the bowel should be left stapled inside the abdominal cavity.

Loop ileostomy should be reserved for obstruction when the tumour is not easily resectable or in case a very abbreviated laparotomy is required. For left-sided lesions in unstable patients, a single-stage procedure represents a time-consuming intervention, at high risk of AL, due to faecal loading and impaired microcirculation induced by sepsis and by the premorbid status of the patient.

Effectiveness of staged procedures (two- or three-step) have been compared in recently published guidelines [1]. In an emergency setting, HP seems suitable for patients who are too unwell to tolerate time-consuming procedures, such as an anastomosis. In fact, HP is a rapid intervention, it minimises surgical trauma it achieves cancer resection, and it eliminates the risk of anastomotic failure. If compared with loop colostomy, HP appears to be associated with shorter overall hospital stay, while perioperative morbidity appears to be the same. Loop colostomy should be reserved for unresectable disease or if neoadjuvant therapy is be planned.

*Statement 5.4: In patient with perforation/obstruction due to colorectal lesions, open abdomen (OA) should be considered if abdominal compartment syndrome is expected; bowel viability should be reassessed after resection. LoE 2-GoRC*

*There is no clear indication to OA in patients with peritonitis. LoE 1-GoR B*

*OA should be closed within 7 days. LoE 1-GoR B*

The OA is defined as the intentional creation of a controlled laparostomy, by leaving the fascial edges of the abdominal wall unapproximated. When used appropriately, this approach is useful in the management of patients at risk of development of abdominal compartment syndrome, or in case the viability of the resected bowel must be reassessed, after an abbreviated laparotomy, before performing an anastomosis. On the contrary, when misused, OA may potentially expose the patient to serious complications, among which the onset of entero-atmospheric fistula is the most worrisome. The inability to re-approximate fascial edges is another drawback of prolonged OA.

In a 1-year series of non-trauma OA described by Bruns et al. [190], the most common preoperative indications for index laparotomy leading to OA were perforated viscus and/or the presence of extra luminal gas on abdominal imaging. Fifty-eight patients received bowel resection at initial operation, and 86% of them were left with intestinal discontinuity at the index operation. DC surgery mandated OA in 37% of cases, while the need for a second look was the indication in 27%; the excessive contamination represented the indication for OA only in 10% of patients. The use of OA in the management of patients with peritonitis is still controversial. Several authors [191, 192] reported no significant differences in morbidity and mortality between on-demand re-laparotomy and planned re-laparotomy groups but showed that on-demand group had shorter ICU and hospital stay. Therefore, peritoneal contamination per se does not represent a strict indication to OA. Aggressive source control followed by abdomen closure should be attempted, and on-demand re-laparotomy should be used instead [193].

In cases when planned re-laparotomy represents a necessity, this should be performed 24–48 h after the initial operation. An abdominal exploration delayed over this period increases the risk of iatrogenic enteric injury, related to intraperitoneal adhesions. The goal to be achieved after OA is the early and definitive closure of the abdominal wall, in order to reduce complications associated to OA. In a systematic review [194], it has been demonstrated that early fascial closure, within 4–7 days of the initial laparotomy, compared to delayed closure was associated with reduced mortality (12.3 versus 24.8%, RR 0.53, *p* < .0001) and complications (RR 0.68, *p* < .0001). In a retrospective review of 42 non-trauma patients, Khan et al. [195]achieved fascial closure within 7 days in 57% of patients, while observing the onset of entero-atmospheric fistula in 4 of 18 patients of the delayed closure group.

*Statement 5.5: A close intraoperative communication between surgeon and anesthesiologist is essential to assess the effectiveness of resuscitation, in order to decide the best treatment option. LoE 2-GoR C*

A uniform approach for critically ill non-trauma patients is crucial to achieve satisfactory outcomes. In terms of decision-making, it is vital to recognise and solve pitfalls in DC or in clinical decision-making. Effective communication and the expression of nontechnical skills among anesthesiologists, nurses and surgeons are essential to manage this typology of patients [196]. It has been demonstrated that failure to communicate critical information in the operating room occurs in approximately 30% of team exchanges [197] and this could lead to inefficiency, emotional tension, delays, resource waste, patient inconvenience, and procedural error, all of which can be detrimental. Similarly, failure to communicate critical information by the anesthesiologist during non-trauma resuscitation of the non-trauma critically ill patient, such as the impairment of metabolic parameters or their improvement achieved by goal-directed resuscitation strategy, may leave the surgeon unaware of the degree of the patient physiologic exhaustion, leading him/her towards wrong surgical decisions. Effective and prompt communication allow the anesthesiologist and the surgeon to recognise potential issues or dangerous circumstances and to adjust their strategies accordingly [198], considering an early DC approach during the multi-faced management of critically ill surgical patients.

## Antibiotic therapy

*Statement 6.1: In patients with colorectal carcinoma obstruction and no systemic signs of infection, antibiotic prophylaxis mainly targeting Gram-negative bacilli and anaerobic bacteria is recommended, because of the potential ongoing bacterial translocation. LoE 1, GoR A*

To establish the effectiveness of antimicrobial prophylaxis for the prevention of surgical wound infection in patients undergoing colorectal surgery, a Cochrane review was published in 2014 including 260 trials and 68 different antibiotics [199].

The review found high-quality evidence, showing that prophylaxis with antibiotics covering aerobic and anaerobic bacteria prior to elective colorectal surgery reduces the risk of surgical wound infection.

Generally, patients with intestinal obstruction with no systemic signs of infections present a risk of surgical site infections similar to patients undergoing elective surgery; in general, antibiotic prophylaxis is sufficient.

A dense population of microorganisms, referred to as the bacterial flora, colonizes the human gastrointestinal tract. Although the gut provides a functional barrier between these organisms and the host, bacterial translocation is a possible event.

Gut translocation of bacteria is defined as the passage of gastrointestinal microflora across the lamina propria to local mesenteric lymph nodes and from there to extranodal sites [200].

Major conditions can contribute to bacterial translocation including a breakdown of the intestinal barrier, an impairment of host immune defense and a loss of the colonisation resistance with bacterial overgrowth in the intestinal tract [201].

Several studies support the concept considering the gut as the source of septic complications; in this sense, bacterial translocation may be an important intermediary mechanism in the development of sepsis [202].

When the mucosa is injured and the intestinal barrier is compromised, a translocation of intestinal microorganisms can occur.

Obstruction cause mucosal injury with a subsequent increase of mucosal permeability and thus bacterial translocation [203, 204].

*Statement 6.2: Prophylactic antibiotics should be discontinued after 24 h (or 3 doses). LoE 1-GoR A.* In these patients, in the light of the need to reduce infections from opportunistic microorganisms—such as *C. difficile*—and to minimise the evolution of multidrug-resistant bacteria, such as ESBL, VRE or KPC, prophylactic antibiotics should be discontinued after 24 h (3 doses) [199].

In 2015, a retrospective review of prospectively collected data on 143 patients with AL after colorectal cancer surgery was published [205]. Of the 143 enrolled patients, 46 (32.2%) were classified in the multidrug-resistant (MDR) group. The use of antibiotics for more than 5 days before diagnosis of AL and diabetes mellitus were identified as independent risk factors of MDR acquisition by multivariate analysis.

*Statement 6.3: In patients with colon carcinoma perforation, antibiotic therapy mainly targeting Gram-negative bacilli and anaerobic bacteria is always suggested. Furthermore, in critically ill patients with sepsis early, use of broader-spectrum antimicrobials is suggested. LoE 1-GoR A*

Antimicrobial therapy, typically empiric antibiotic treatment, plays an important role in the management of colon cancer perforation. Initial antimicrobial therapy for patients with IAI is empiric in nature because patients need immediate treatment and microbiological data (culture and susceptibility results) usually requires ≥ 24–48 h for the identification of pathogens and patterns of antibiotic susceptibility [206].

The empirically designed antimicrobial regimen depends on the pathogens presumed to be involved, the risk factors indicative of major resistance patterns and the underlying severity of infection.

Considering the intestinal microbiota of the large bowel, patients with colon cancer perforations require antibiotic coverage for Gram-negative bacteria, as well as for anaerobes.

The virulent microorganisms in colorectal procedures are derived from the bowel lumen, where there are high concentrations of bacteria, such as *B. fragilis* and other obligate anaerobes and *Enterobacteriaceae* including *E. coli* [207].

The choice of the antimicrobial regimen poses serious problems for the management of unstable patients with sepsis. In these patients, an early and appropriate empirical antimicrobial therapy has a significant impact on the outcome [208]. Therefore, in these patients, early use of broad-spectrum intravenous antimicrobials is always suggested.

*Statement 6.4: In patients with perforated colorectal cancer, antibiotic therapy should consider bacterial resistance and should be refined according to the microbiological findings, once available. LoE 1-GoR B*

The vast majority of colon cancer perforations represent community-acquired infections. The main resistance threat in these IAI is posed by extended-spectrum beta-lactamase (ESBL) producing *Enterobacteriaceae*, which are becoming increasingly common in community-acquired infections worldwide [209].

The results of microbiological testing may have great importance for the choice of therapeutic strategy of every patient, in particular in the rationalisation of targeted antimicrobial treatment [206].

The duration of antibiotic therapy is a matter of debate, usually ranging from 4 to 7 days according to clinical features (source control, fever, leukocytosis, C-reactive protein, procalcitonin) [207, 210].

## Conclusions: grey areas and opportunities for improvements

We found some limitations within the present guidelines:

- They fail to cover all the possible abdominal scenarios when colon cancer occurs as an emergency: for example, associated resections were not taken into considerations, neither we discussed about therapeutic strategies in case of evidence of peritoneal carcinomatosis.
- Despite our attempts to underline suggestions in case of low technical resources, the present guidelines are generally oriented toward hospitals with high level of resources.

On the other side, in our opinion, the current guidelines suggest some stimuli for doctors involved in this field:

- To review the approach to patient suffering from abdominal pain by introducing and promoting the use of bedside abdominal US.
- To bear in mind that the emergency surgeon should have a strong oncologic background or that the specialised colorectal surgeon should have a strong background of surgical pathophysiology, emergency surgery and damage control philosophy.
- To promote the use of clinical pathways within singular Hospitals.

All the considerations mentioned above, and further by readers, will be an incentive for further revisions and improvements.

## Figures and Tables

**Fig. 2:**
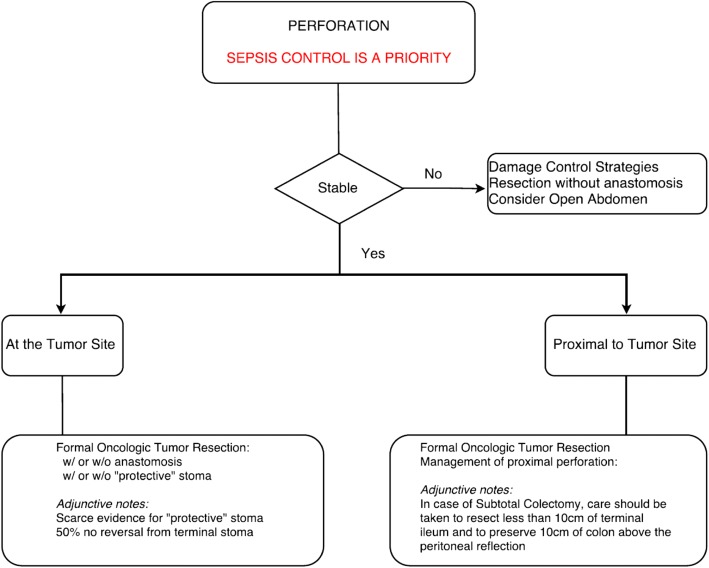
Flowchart for the management of colonic perforation due to colorectal cancer

**Fig. 3:**
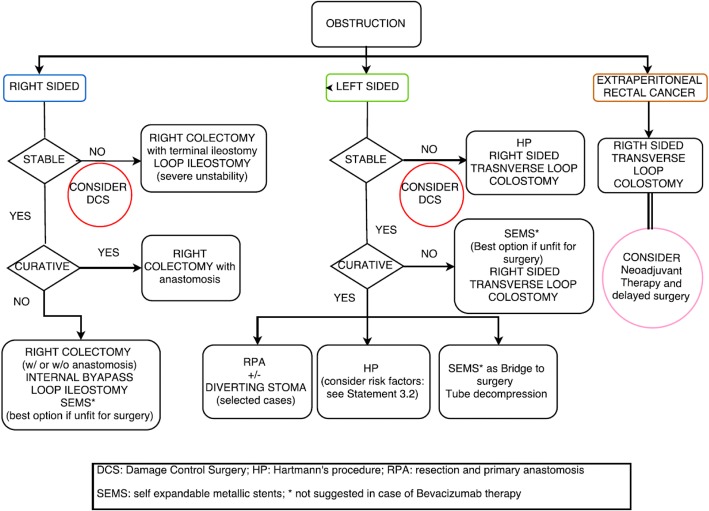
Flowchart for the management of colonic obstruction due to colorectal cancer

## Appendix 1

**Table 6:** Table of statements

Topic	No.	LoE	GoR	
1. Diagnosis	1.1	3	B	The clinical presentation is variable, except for lower rectal cancer, in which case digital examination could be diagnostic. Laboratory tests are not specific. Clinical evaluation and laboratory tests have high variability and low specificity; therefore, the escalation to further diagnostic tools, whenever available, is mandatory.
	1.2	3	B	(a) In case of clinical suspicion of colon obstruction, computed tomography (CT) scan achieves the confirmation of diagnosis better than abdominal ultrasound (US), which performs better than abdominal plain X-ray. If CT scan is not available, a water-soluble colonic contrast enema is a valid alternative in for identifying the site and the nature of obstruction. (b) In case of clinical suspicion of perforation, abdominal CT scan, which performs better than abdominal US, should achieve diagnostic confirmation. US performs better than abdominal plain X-ray. LoE 3, GoR B.
	1.3	3	B	In stable patients, direct visualisation of the site of colonic obstruction should be considered when colonoscopy is available. In this situation, biopsies should be obtained, especially when the deployment of an endoscopic stent is planned. LoE 3, GoR B.
	1.4	3	B	In case of clinical suspicion of perforation, abdominal CT scan, which performs better than abdominal US, should achieve diagnostic confirmation. US performs better than abdominal plain X-ray.
	1.5	3	B	There is no specific data regarding staging pathways of CRC presenting as an emergency. CT scan performs better than US in the abdomen and should be suggested for staging in the suspicion of cancer-related colorectal emergencies. CT scan of the thorax is not strictly recommended. LoE 3, GoR B
	1.6	3	B	There is no specific data regarding staging pathways of CRC presenting as emergency. CT scan performs better than US in the abdomen and should be suggested for staging in the suspicion of cancer-related colorectal emergencies. CT scan of the thorax is not strictly recommended.
2. Perforation	2.1	2	B	When diffuse peritonitis occurs in cancer-related colon perforation, the priority is the control of the sepsis source of sepsis. Prompt combined medical treatment is advised. LoE 2, GoR B
	2.2	3	B	Oncologic resection should be performed in order to obtain better oncologic outcomes. • Perforation at the tumour site: formal resection with or without anastomosis, with or without stoma • Perforation proximal to tumour site (diastasic): simultaneous tumour resection and management of proximal perforation is indicated. Depending on the colonic wall conditions, a subtotal colectomy may be required. The surgeon should consider that only a small proportion of patients undergo reversal of terminal stoma.
3. Left colon obstruction	3.1	2	B	Loop colostomy (C) versus Hartmann’s procedure (HP). Hartmann’s procedure should be preferred to simple colostomy, since colostomy appears to be associated with longer overall hospital stay and need for multiple operations, without a reduction in perioperative morbidity LoE 2, GoR B. Loop colostomy should be reserved for to unresectable tumours (if SEMS is not feasible), for severely ill patients who are too unfit for major surgical procedures or general anaesthesia.
	3.2	3	B	Hartmann’s procedure (HP) versus resection and primary anastomosis (RPA) RPA should be the preferred option for uncomplicated malignant left-sided large bowel obstruction in absence of other risk factors. Patients with high surgical risk are better managed with HP.
	3.3	4	C	RPA: the role of diverting stoma There is no evidence supporting that a covering stoma can reduce the risk of anastomotic leak and its severity.
	3.4	2	B	Total colectomy versus segmental colectomy. In absence of caecal tears/perforation or, evidence of bowel ischemia or synchronous right colonic cancers, total colectomy should not be preferred to segmental colectomy, since it does not reduce morbidity and mortality and is associated with higher rates of impaired bowel function. LoE 2, GoR B.
	3.5	2	B	Intraoperative colonic irrigation (ICI) versus manual decompression (MD) ICI and MD are associated with same mortality/morbidity rate. The only significant difference is that MD is a shorter and simpler procedure. Either procedure could be performed, depending on the experience/preference of the surgeon.
	3.6	4	C	RPA: the role of laparoscopy. The role use of laparoscopy in the emergency treatment of OLCC cannot be recommended and should be reserved to selected favourable cases and in specialised centers.
	3.7	4	C	Tube decompression (TD) TD can be a valid alternative option as BTS for high-risk OLCC.
	3.8	3	B	Palliation: SEMS versus colostomy. In facilities with capability for stent placement, SEMS should be preferred to colostomy for palliation of OLCC since it is associated with similar mortality/morbidity rates and shorter hospital stay. LoE 1-GoR A. Alternative treatments to SEMS should be considered in patients eligible for to a bevacizumab-based therapy. Involvement of the oncologist in the decision is strongly recommended. LoE 3-GoR B
	3.9	1	B	Bridge to surgery (BTS): SEMS and planned surgery versus emergency surgery SEMS as bridge to elective surgery offers a better short-term outcome than direct emergency surgery. The complications are comparable, but the stoma rate is significantly smaller. Long-term outcomes appear comparable as well, but evidence remains suboptimal; further studies are necessary. For these reasons, SEMS as BTS cannot be considered the treatment of choice in the management of OLCC, whilst it may represent a valid option in selected cases and in tertiary referral hospitals.
	3.10	1	A	Extraperitoneal rectal cancer. Locally advanced rectal cancers are better cured treated with a multimodal approach including neoadjuvant chemoradiotherapy. LoE 1-GoR A. In case of acute obstruction, resection of the primary tumour should be avoided and a stoma should be fashioned, in order to permit a correct staging and a more appropriate oncologic treatment. Transverse colostomy seems to be the best option, but other modalities can be considered. SEMS is not indicated.
4. Right occlusion	4.1	2	B	In case of right-sided colon cancer causing acute obstruction, right colectomy with primary anastomosis is the preferred option. A terminal ileostomy associated with colonic fistula represents a valid alternative when if a primary anastomosis is considered unsafe. LoE 2-GOR B
	4.2	2	B	For unresectable right-sided colon cancer, a side-to-side anastomosis between the terminal ileum and the transverse colon (the internal bypass) can be performed; alternatively, a loop ileostomy can be fashioned. Decompressive caecostomy should be abandoned.
	4.3	4	B	SEMS as bridge to elective surgery for ORCC is not recommended. It may represent an option in high-risk patients.
	4.4	3	B	In a palliative setting, SEMS can be an alternative to emergency surgery (ES) in for obstruction due to right colon cancer obstruction. LoE 3, GOR B
5. Unstable patients	5.1	2	C	A patient with perforation/obstruction due to colorectal cancer should be considered unstable and therefore amenable for damage control treatment, if at least one of the following items is present: ● pH < 7.2 ● Core temperature < 35 °C ● BE < − 8 ● Laboratory/clinical evidence of coagulopathy ● Any signs of sepsis/septic shock, including the necessity of inotropic support.
	5.2	2	C	Damage control should be started as soon as possible, in rapid sequence after resuscitation.
	5.3	2	C	If the patient is unstable, definitive treatment can be delayed. Right-sided obstruction: Right colectomy with terminal ileostomy should be considered the procedure of choice. Severely unstable patients should be treated with a loop ileostomy. Right-sided perforation: Right colectomy with terminal ileostomy should be considered the procedure of choice. If an open abdomen has to be considered, the stoma creation should be delayed. Right colectomy with ileo-colic anastomosis could be performed if no significant increase in operative time is required and good bowel vascularisation is present and expected in the perioperative time. Left-sided obstruction: Hartmann’s procedure should be considered the procedure of choice. Severe unstable patients should be treated with a loop transverse colostomy. Left-sided perforation: Hartmann’s procedure should be considered the procedure of choice. If an open abdomen has to be considered, the stoma creation should be delayed.
	5.4	2	C	In patient with perforation/obstruction due to colorectal lesions, open abdomen (OA) should be considered if abdominal compartment syndrome is expected; bowel viability should be reassessed after resection. There is no clear indication to OA in patients with peritonitis. OA should be closed within 7 days.
	5.5	2	C	A close intraoperative communication between surgeon and anesthesiologist is essential to assess the effectiveness of resuscitation, in order to decide the best treatment option.
6. Antibiotic therapy	6.1	1	A	In patients with colorectal carcinoma obstruction with no systemic signs of infection, antibiotic prophylaxis is recommended.
	6.2	1	A	Prophylactic antibiotics should be discontinued after 24 h (or 3 doses).
	6.3	1	B	In patients with intestinal obstruction, even without systemic signs of infections, antibiotic prophylaxis mainly targeting Gram-negative bacilli and anaerobic bacteria is suggested, because of the potential ongoing bacterial translocation.
	6.4	1	A	In patients with colon carcinoma perforation, antibiotic therapy mainly targeting Gram-negative bacilli and anaerobic bacteria is always suggested. Furthermore, in critically ill patients with sepsis early, use of broader-spectrum antimicrobials is suggested.
	6.5	1	B	In patients with perforated colorectal cancer, antibiotic therapy should consider bacterial resistance, and should be refined according to the microbiological findings, once available.

## Abbreviations

AL: Anastomotic leak
BTS: Bridge to surgery
CRCE: Colon Rectal Cancer Emergencies
CT scan: Computed Tomography
DC: Damage control
DFS: Disease-free survival
ES: Emergency surgery
GoR: Grade of recommendation
HP: Hartmann’s procedure
IAI: Intra-abdominal infection
ICI: Intraoperative colonic irrigation
LBO: Large bowel obstruction
LoE: Level of evidence
MD: Manual decompression
OLCC: Obstructed left colon cancer
ORCC: Obstructed right colon cancer
OS: Overall survival
RCT: Randomised clinical trial
RPA: Resection and primary anastomosis
SEMS: Self-expanding metallic stent
US: Ultrasound
WSES: World Society of Emergency Surgery
